# Repeated Cocaine Intake Differentially Impacts Striatal D_2/3_ Receptor Availability, Psychostimulant-Induced Dopamine Release, and Trait Behavioral Markers of Drug Abuse

**DOI:** 10.3390/ijms241713238

**Published:** 2023-08-26

**Authors:** Ginna Urueña-Méndez, Andrea Dimiziani, Lidia Bellés, Raphaël Goutaudier, Nathalie Ginovart

**Affiliations:** 1 Department of Psychiatry, Faculty of Medicine, University of Geneva, 1211 Geneva, Switzerland; ginna.uruenamendez@unige.ch (G.U.-M.); belleslidia@gmail.com (L.B.); raphael.goutaudier@unige.ch (R.G.); 2 Department of Basic Neurosciences, Faculty of Medicine, University of Geneva, 1211 Geneva, Switzerland

**Keywords:** dopamine, D_2/3_ receptors, impulsivity, novelty-seeking, drug abuse, addiction

## Abstract

Current research indicates that altered dopamine (DA) transmission in the striatum contributes to impulsivity and novelty-seeking, and it may mediate a link concerning a higher susceptibility to drug abuse. Whether increased susceptibility to drug abuse results from a hyperdopaminergic or hypodopaminergic state is still debated. Here, we simultaneously tracked changes in DA D_2/3_ receptor (D_2/3_R) availability and amphetamine-(AMPH)-induced DA release in relation to impulsivity and novelty-seeking prior to, and following, cocaine self-administration (SA) in Roman high- (RHA) and low- (RLA) avoidance rats. We found that high-impulsive/high novelty-seeking RHA rats exhibited lower D_2/3_R availabilities and higher AMPH-induced DA release in the striatum that predicted higher levels of cocaine intake compared with RLAs. Cocaine SA did not alter striatal D_2/3_R availability or impulsivity in RHA or RLA rats. Critically, cocaine exposure led to a baseline-dependent blunting of stimulated DA release in high-impulsive/high novelty-seeking RHA rats only, and to a baseline-dependent increase in novelty-seeking in low-impulsive/low novelty-seeking RLA rats only. Altogether, we propose that susceptibility to drug abuse results from an innate hyper-responsive DA system, promoting impulsive action and novelty-seeking, and producing stronger initial drug-reinforcing effects that contribute to the initiation and perpetuation of drug use. However, with repeated cocaine use, a tolerance to drug-induced striatal DA elevations develops, leading to a compensatory increase in drug consumption to overcome the reduced reward effects.

## 1. Introduction

Understanding why only a subset of individuals initially use drugs recreationally, before progressing to drug abuse and dependence, remains a major challenge in addiction research. Besides the contribution of genetic and environmental risk factors [1], personality traits concerning impulsivity and novelty (or sensation) seeking are widely considered to contribute to the development of substance use disorders (SUD), due to their high prevalence in drug abusers [2,3,4,5], and their prospective association in adolescents who later misuse substances [6,7,8,9,10]. However, cross-sectional studies indicate that impulsivity, but not novelty-seeking, is elevated in non-substance-dependent siblings of substance-dependent users [5,11], suggesting that these two personality traits may differentially predict the risk of SUD. There is also evidence to suggest that impulsivity and novelty-seeking may result from drug use [12,13], which has cast a shadow on the role of these traits as risk factors for SUD [13,14,15]. Studies performed in rodents to disambiguate whether impulsivity and novelty-seeking are causes or consequences of drug abuse have produced mixed results. Rats with high impulsivity, as identified by enhanced premature responding during behavioral inhibition tasks, exhibit increased rates of cocaine [16,17], nicotine [18], and alcohol [19] self-administration (SA), indicating that impulsive actions predics drug intake, although other studies failed to replicate this finding [20,21,22,23,24]. Moreover, the effect of drug exposure on impulsive action in rodents is unclear, with some studies showing either no alteration [25,26,27] or a worsening [24,28,29,30] in impulsivity. Intriguingly, some data suggest that the effect of drug intake on impulsive action may be baseline-dependent. However, here again, the direction of change is unclear, with studies showing a reduction in premature responses, but only in high-impulsive animals [16,21], whereas other reported an increase, but only in low-impulsive animals [22]. Less attention has been paid to the predictive value of novelty-seeking, as assessed by the preference for a novel environment in a free-choice procedure, on drug abuse. Although some studies showed that novelty-seeking predicts higher levels of drug use [31,32], other studies failed to replicate this finding [33,34,35], but suggest that novelty-seeking rather predicts the transition from controlled to compulsive drug use [34]. To our knowledge, the effect of drug exposure on novelty-seeking remains untested. Thus, the relationship between novelty-seeking, and to a lesser extent impulsive action, and the propensity to use drugs is unclear, as are the effects of repeated drug exposure on these behavioral traits.

Converging evidence indicates that impulsivity and novelty-seeking share overlapping neural circuits with those implicated in drug abuse, and that all three are strongly linked to alterations in dopaminergic functioning in the striatum [36,37,38]. Self-reported impulsivity [39,40], impulsive actions [41,42], and novelty-seeking [43] have all been related to striatal deficits in dopamine (DA) D_2/3_ receptors (D_2/3_R) in healthy individuals. In addition to D_2/3_R deficits, both motor impulsive individuals [44,45] and novelty-seekers [46,47] also exhibit a heightened release of striatal DA in response to psychostimulants, an effect that has been related to the subjective “high” and euphoria experienced by healthy subjects to the drug [48,49]. Preclinical studies have broadly confirmed these human findings, as both impulsive action and novelty-seeking in rodents have been related to reduced D_2/3_R availabilities [16,50,51,52] and to a heightened psychostimulant-induced DA release [52] in the striatum. These findings linking impulsivity and novelty-seeking to increased pre-synaptic and decreased post-synaptic markers of striatal DA function conflict with observations in drug abusers. Indeed, neuroimaging studies have consistently shown that both pre-synaptic and post-synaptic indices of striatal DA function are down-regulated in drug abusers, as revealed by the reduced availabilities of D_2/3_R, and a diminished amphetamine-induced DA release [53,54]. Here again, it is difficult to disentangle whether these alterations represent pre-existing factors or consequences of drug abuse, as some studies indicate that chronic psychostimulant exposure reduced striatal D_2/3_R availability [55,56,57] and amphetamine-induced DA release [58,59] in rodents. However, not all studies support these findings [60,61]. Critically, baseline differences in impulsive actions may be important for this effect, as one study showed that cocaine SA increased, rather than reduced, striatal D_2/3_R availability, but only in high-impulsive animals [21]. This raises the important point that drug exposure may produce differential, baseline-dependent effects on striatal DA markers and on behavioral traits linked to drug abuse, an aspect that has been scarcely considered so far and that may explain some of the inconsistencies in the literature.

Thus, there is still a need for longitudinal studies to identify which traits are predisposed to, and which result from, drug use, especially when considering baseline levels. There is also a need to investigate how pre- and post-synaptic markers of striatal DA function predict drug abuse and how they are affected by drug exposure, possibly in a baseline-dependent manner. In the present study, we therefore investigated the consequences of repeated cocaine exposure on impulsive action, novelty-seeking, and in vivo measures of pre- and post-synaptic markers of striatal DA function. Impulsive action and novelty preference were evaluated using the five-serial reaction time task (5-CSRTT) and the novelty-induced place preference (NIPP) test, respectively. We used single photon emission computed tomography (SPECT) imaging and the D_2/3_R radiotracer, [^123^I]IBZM, to concurrently assess D_2/3_R availability and amphetamine-induced DA release in the dorsal and ventral striatum. All measurements occurred prior to, and following, a regimen of cocaine or saline self-administration, using a within-subject repeated measures design. To further assess the potential baseline-dependent effects of cocaine exposure on both behavioral traits and on DA variables, we used the Roman high-(RHA) and Roman low-avoidance (RLA) rat lines, which display divergent phenotypes in terms of impulsive action, novelty-seeking, striatal DA function, propensity to self-administer cocaine, and to relapse [52,62,63].

## 2. Results

### 2.1. Behavioral Phenotype and D_2/3_R-Related Signaling at Baseline in RHA and RLA Rats

**Figure 1:**
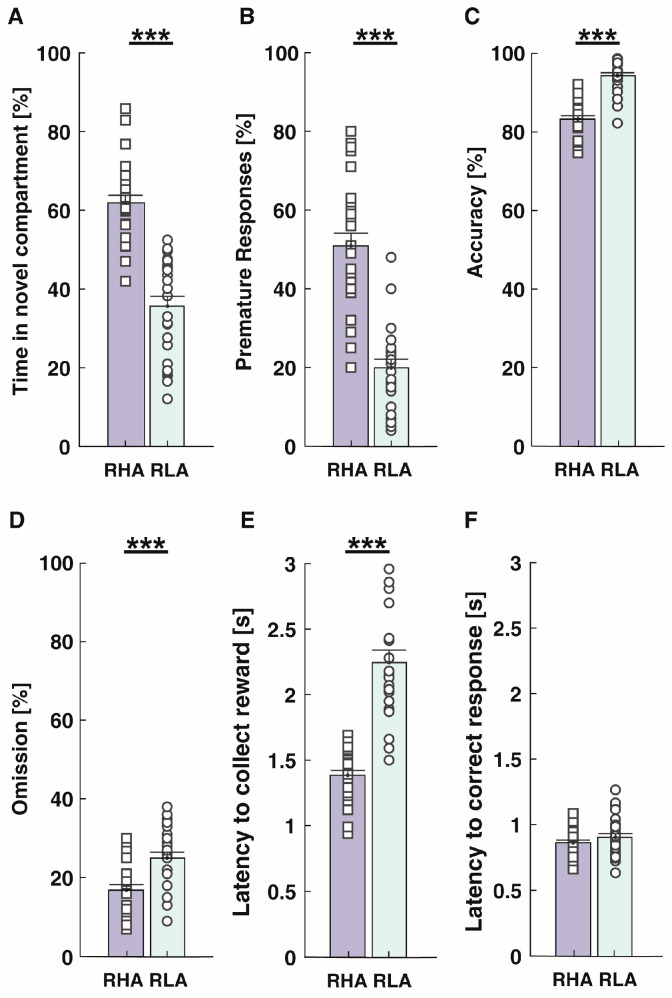
Behavioral phenotypes of RHA and RLA rats at baseline (*n* = 27/line). (**A**) Novelty-seeking, as assessed by the percentage of time spent in a novel vs. familiar compartment in the novelty-induced preference test, was greater in RHA than in RLA rats. (**B**) Impulsive action, as assessed by the percentage of premature responses in the 5-CSRTT, was higher in RHA than in RLA rats. Other behavioral measures in the 5-CSRTT included the percentage accuracy (**C**), percentage of omissions (**D**), the latency to collect reward (**E**), and the latency to correct response (**F**). Data appear as mean ± SEM. Significantly different in RHA vs. RLA rats at *** *p* < 0.001.

The behavioral performances of RHA and RLA rats during the NIPP test and 5-CSRTT are shown in Figure 1. In accordance with a recent study [52], RHA rats displayed a higher preference towards the novel compartment in the NIPP test (Mann–Whitney U = 23, *p* < 0.001; Figure 1A), and committed to more premature responses (*t*-test = 7.95, *p* < 0.001) than RLAs in the 5-CSRTT (Figure 1B), indicating a greater novelty preference and greater impulsivity. During the 5-CSRTT, RHA rats also displayed a lower response accuracy than RLAs (Mann–Whitney U = 30, *p* < 0.001; Figure 1C), suggesting reduced attentional processing in RHA rats. Consistent with previous reports [64,65], accuracy was inversely correlated with premature responses (r = −0.75; *p* < 0.001). RHAs also committed to fewer omissions than RLAs (*t*-test = −3.89, *p* < 0.001; Figure 1D), and latency to collect rewards was higher in RLAs than in RHAs (*t*-test = −9.16, *p* < 0.001; Figure 1E), suggesting that RHA rats were more motivated to the task. The latency to correct responses were similar in RHA and RLA rats (*t*-test = −1.14, *p* = 0.25; Figure 1F), indicating that the processing speed and motor ability were similar in both Roman rat lines. The total number of trials completed summed-up to 100 trials in both lines.

**Figure 2:**
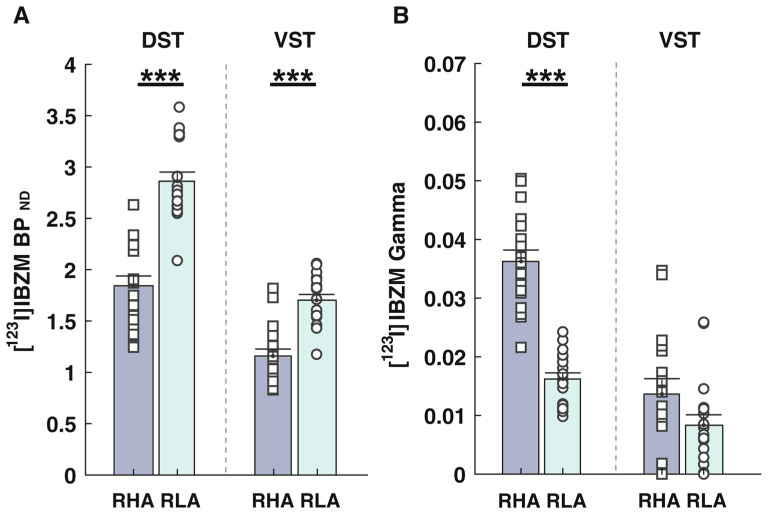
D_2/3_R-related signaling in RHA and RLA rats at baseline (n = 18 rats/line)**.** (**A**) D_2/3_ receptor availabilities, as indexed by the non-displaceable binding potential (BP_ND_) of the D_2/3_ receptor antagonist radiotracer [^123^I]IBZM, was lower in both the dorsal (DST) and the ventral (VST) striatum of RHA rats, as compared with RLA rats. (**B**) AMPH-induced dopamine release, as indexed by [^123^I]IBZM Gamma values, was higher in RHA rats compared with RLA rats. Data appear as mean ± SEM. A significantly different in RHA vs. RLA rats at *** *p* < 0.001.

Baseline levels of D_2/3_R availabilities, as indexed by [^123^I]IBZM BP_ND_, were significantly lower in both the DST (*t*-test = −7.75, *p* < 0.001) and VST (*t*-test = −6.17, *p* < 0.001) of RHA rats compared with RLA rats (Figure 2A). BP_ND_ values the DST and VST were estimated with very good accuracy, as evidenced by the low mean percentage standard error (%SE) of the parameter estimates obtained during nonlinear least square fittings of [^123^I]IBZM kinetics (Table S1). The mean %SE values were less than 4%, indicating that BP_ND_ was very well identified in both striatal subdivisions. AMPH-induced DA release, as indexed by [^123^I]IBZM Gamma, was significantly higher in the DST of RHA rats when compared with RLA rats (*t*-test = 9.11, *p* < 0.001; Figure 1B). However, as previously observed [32], although [^123^I]IBZM gamma in DST was estimated with reasonable precision, with %SE values lower than 25%, [^123^I]IBZM Gamma in VST showed poor identifiability with %SE values exceeding 100% in both lines (Table S1). [^123^I]IBZM Gamma in VST was thus treated as unreliable and excluded from further analysis.

### 2.2. Intravenous Cocaine Self-Administration

**Figure 3:**
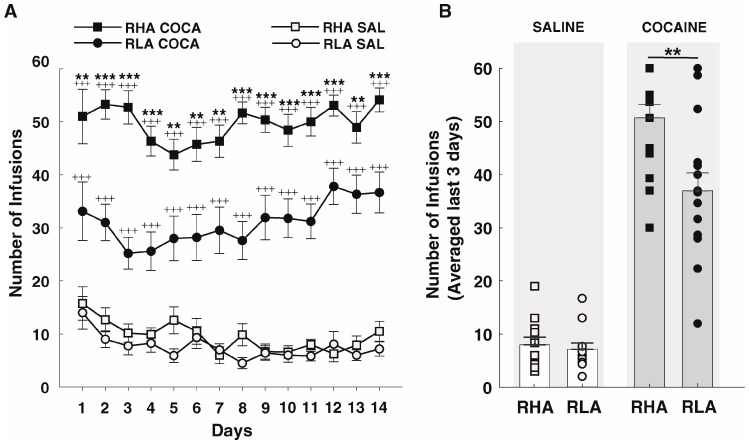
Differences in intravenous Cocaine Self-Administration (SA) in RHA and RLA rats. (**A**) Number of infusions during the 14 days of SA testing. (**B**) Average numbers of infusions earned during the last three sessions of SA. When exposed to cocaine (n = 15/line), RHA rats self-injected significantly more cocaine than RLA rats during all SA sessions. In contrast, when exposed to the saline-vehicle (n = 12/line), both lines displayed similarly low levels of infusions during SA testing. Data appear as mean ± SEM. A significantly different result in RHA vs. RLA rats is indicated by ** *p* < 0.01 and *** *p* < 0.001, and in Cocaine vs. Saline, it is indicated by +++ *p* < 0.001.

High-impulsive RHA rats clearly exhibited a higher rate of cocaine SA than non-impulsive RLAs (Figure 3A). The number of cocaine infusions gained on each SA session was significantly higher in RHAs than in RLAs (*p* < 0.01 in all sessions, Mann–Whitney U-tests; Figure 3A). In contrast, no significant differences were observed in saline self-administration between the two lines (*p* > 0.05 in all sessions, Mann–Whitney U-tests; Figure 3A). GLM ANOVA showed the main effects of treatment (F_1,50_ =  267.9, *p* < 0.001), line (F_1,50_ = 6.8, *p* = 0.01), and a treatment by line interaction effect (F_1,50_ =  4.23, *p* < 0.05) on the average number of infusions earned across the last three days of SA (Figure 3B). Post hoc analyses confirmed that although RHA and RLA rats did not differ the number of saline infusions (*p* = 0.68), RHAs self-administered significantly more cocaine infusions than RLAs during the last 3 days of testing (*p* = 0.003).

### 2.3. Effects of Cocaine SA on Impulsivity and Novelty-Seeking

**Figure 4:**
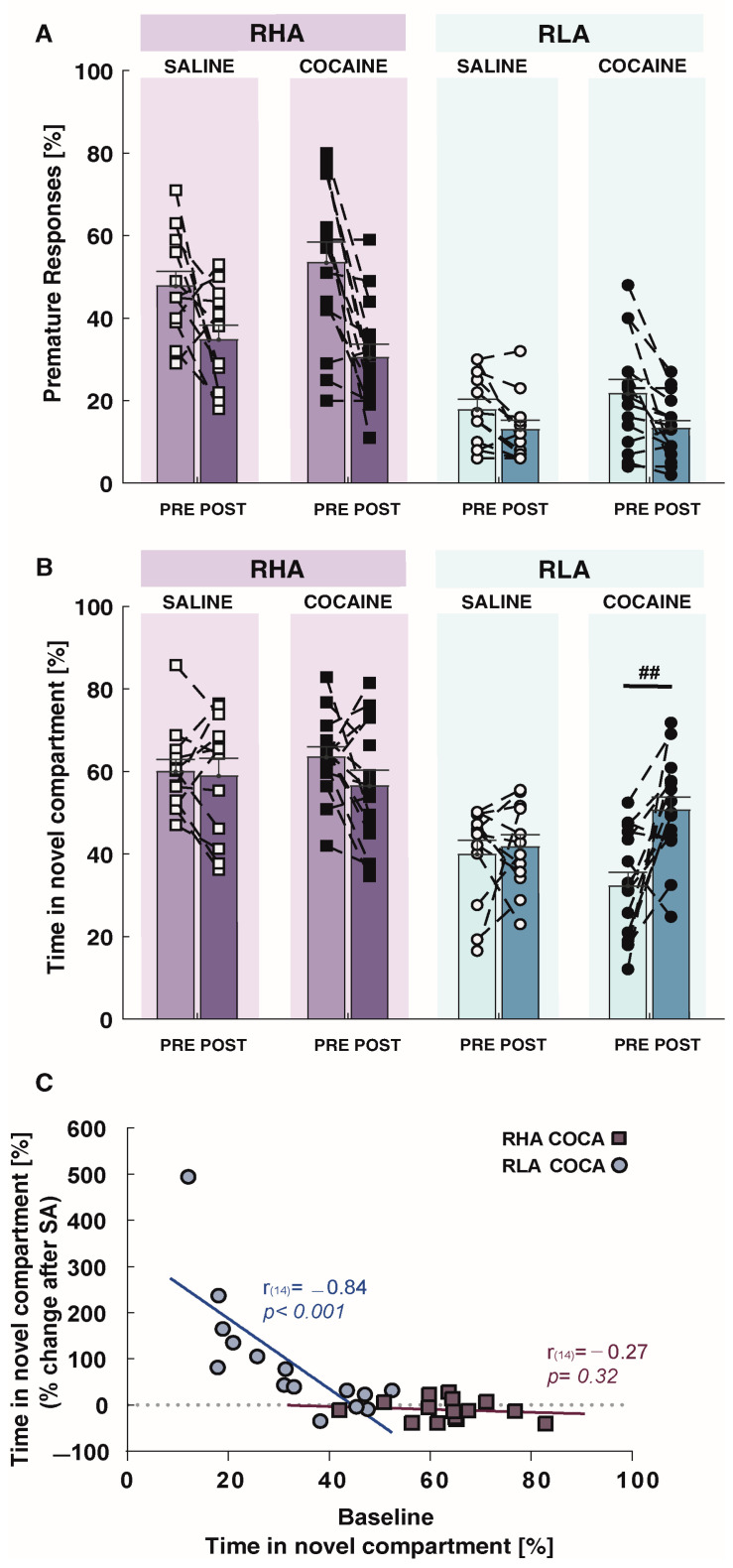
Behavioral effects of the cocaine SA on impulsivity and novelty preference in RHA and RLA rats. (**A**) Impulsive action decreased in both RHAs and RLAs, regardless of prior cocaine or saline self-administration (SA). (**B**) Novelty-seeking was not affected by a history of cocaine or saline SA in RHA rats, but it was significantly increased in RLA rats following cocaine but not saline SA. (**C**) The effect of cocaine on novelty-seeking was baseline-dependent in RLA rats but not in RHA rats. So that the lower levels of novelty-seeking that were found at baseline (i.e., pre-cocaine) predicted a higher cocaine-induced increase in novelty-seeking. Data appear as mean ± SEM, significantly different at ## *p* < 0.01. in pre vs. post cocaine SA. Cocaine groups, n = 15/line. Saline groups, n = 12/line.

RM-GLM ANOVA revealed a main effect of time (F_1,50_ = 34.75, *p* < 0.001) and line (F_1,50_ = 85.52, *p* < 0.001), but not main effect of treatment (F_1,50_ = 0.255, *p* = 0.616) on premature responses (Figure 4A; Table 1). Importantly, no significant interaction between factors was revealed (time x treatment x line: F_1,50_ = 0.580, *p* = 0.450). Hence, premature responses were significantly reduced from pre- to post-SA measurement, irrespective of the line or the treatment (i.e., cocaine or saline). These results indicate a possible learning effect, with an improvement in premature responses likely resulting from the repetition of 5-CSRTT testing during the post-SA session. Cocaine exposure had no effect on the other parameters of the 5-CSRTT, except for an increase in the response accuracy in RHA rats (*t*-test = −3.18, *p* < 0.01; Table 1).

RM-GLM ANOVA showed a significant three-way interaction between time, treatment, and line (F_1,50_ = 7.36, *p* = 0.009) on novelty-seeking (Figure 4B; Table 1). Paired post hoc comparisons of the pre- to post-SA measurement showed that cocaine SA significantly increased novelty-seeking in RLAs (*p* = 0.002), but not in RHAs (*p* = 0.09). Furthermore, a strong relationship was observed between the percentage changes in novelty-seeking following cocaine exposure and baseline novelty-seeking in RLA (r = 0.87, *p* < 0.001; Figure 4C) but not in RHA rats (r = −0.27, *p* = 0.32; Figure 4C). An inspection of the data indicated the presence of an outlier in the RLA cocaine–SA group (Figure 4C). After the removal of this outlier, the correlation between the RLAs still remained highly significant (r = 0.84, *p* < 0.001). No significant effect of the saline SA on novelty-seeking was found in either RHAs or RLAs.

### 2.4. Effects of Cocaine on Striatal D_2/3_R Availabilities and AMPH-Induced DA Release

**Figure 5:**
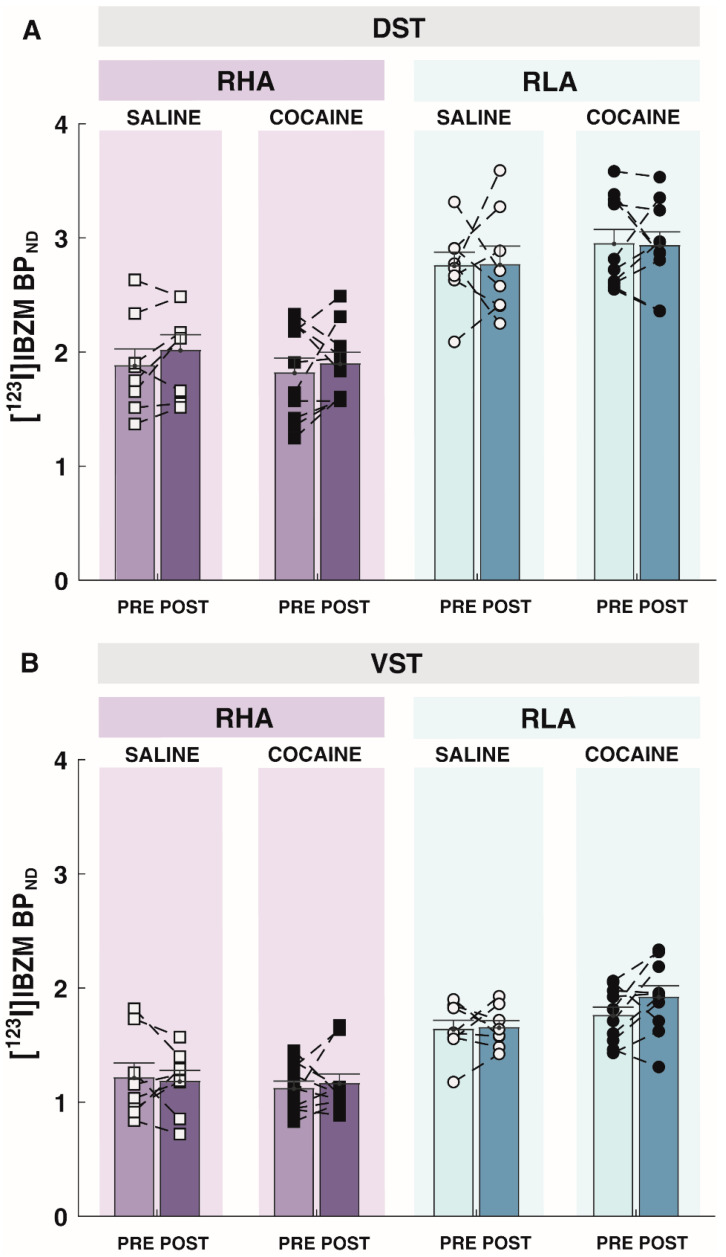
Effects of cocaine self-administration on D_2/3_ receptor availabilities, as indexed by the non-displaceable binding potential (BP_ND_) of [^123^I]IBZM in (**A**) the dorsal striatum (DST) and (**B**) the ventral striatum (VST). As with saline exposure (n = 8/line), prior cocaine exposure (n = 10/line) had no significant effect on [^123^I]IBZM BP_ND_ in DST or VST. Data appear as mean ± SEM.

The analysis of [^123^I]IBZM BP_ND_ as an index of D_2/3_R availabilities in DST (Figure 5A) and VST (Figure 5B) revealed a main effect of line (DST: F_1,32_ = 65.80, *p* < 0.001; VST: F_1,32_ = 56.02, *p* < 0.001), but not main effect of treatment (DST: F_1,32_ = 0.15, *p* = 0.704; VST: F_1,32_ = 0.82, *p* = 0.372) and time (DST: F_1,32_ = 0.71, *p* = 0.41; VST: F_1,32_ = 1.02, *p* = 0.319). There was no interaction between time, line, and treatment on [^123^I]IBZM BP_ND_ in either the DST (F_1,32_ = 0.013, *p* = 0.91) or the VST (F_1,32_ = 0.142, *p* = 0.71). RHA rats displayed persistently lower levels of striatal D_2/3_R availabilities compared with RLAs both at baseline and following cocaine exposure, and neither saline nor cocaine SA affected D_2/3_R availabilities in the DST or the VST of RHA or RLA rats.

**Figure 6:**
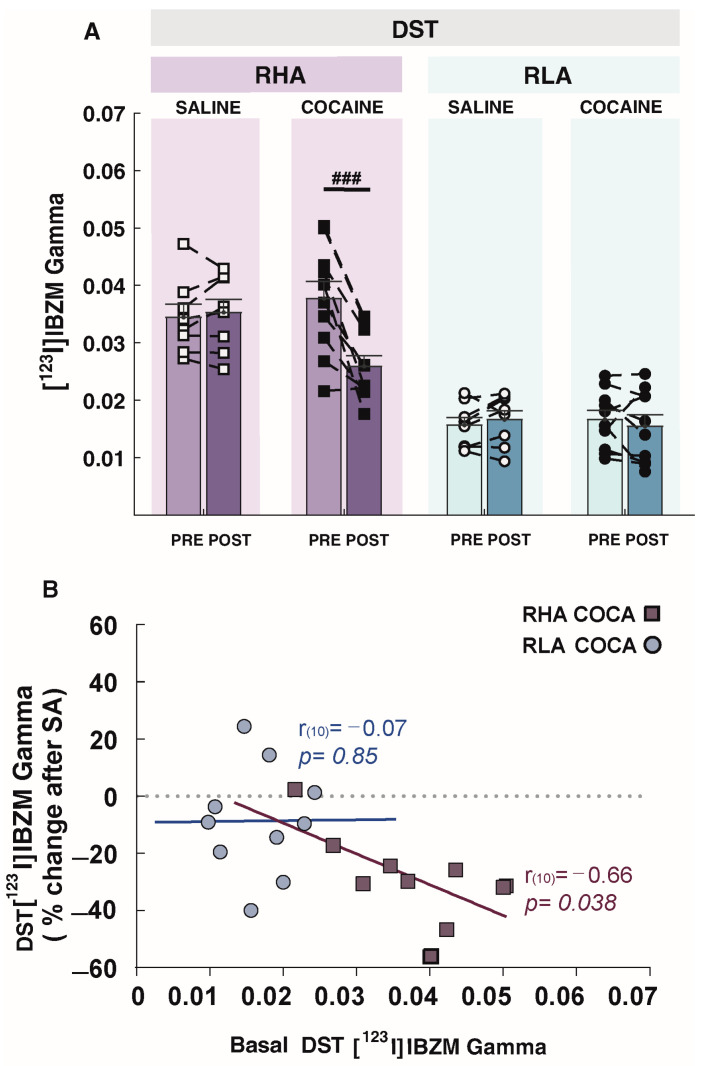
Effects of cocaine self-administration (SA) on [^123^I]IBZM Gamma values as an index of amphetamine (AMPH)-induced dopamine (DA) release in RHA and RLA rats. (**A**) AMPH-induced DA release in the DST significantly decreased after cocaine, but not saline, self-administration (SA) in RHA rats. No changes occurred in RLA rats after either saline or cocaine SA. (**B**) The effect of cocaine on DA release in the DST was baseline-dependent exclusively in RHA rats, so that a higher AMPH-evoked DA release at baseline (i.e., pre-cocaine) predicted a higher decrease in AMPH-induced DA release following cocaine SA. Data appear as mean ± SEM, significantly different at ### *p* < 0.001 concerning pre vs. post cocaine intake. Cocaine groups, n = 10/line. Saline groups, n = 8/line.

In contrast, the analysis of DST [^123^I]IBZM Gamma as an index of AMPH-induced DA release demonstrated a main effect of time (F_1,32_ = 14.33, *p* = 0.001), line (F_1,32_ = 79.19, *p* < 0.001), and a significant time by treatment by line interaction (F_1,32_ = 10.86, *p* = 0.002). Post hoc contrasts indicated that at baseline, RHAs displayed higher levels of AMPH-induced DA release in DST than RLAs (*p* < 0.001; Figure 6A). Paired post hoc contrasts of the pre- to post-SA measurement showed that cocaine SA significantly decreased DST Gamma in RHAs (*p* < 0.001), but not in RLAs (*p* = 0.198). Moreover, a significant inverse relationship was observed between the percentage changes in DST [^123^I]IBZM Gamma following cocaine exposure and baseline measures of [^123^I]IBZM Gamma in RHA (r = 0.66, *p* = 0.038; Figure 6B), but not in RLA rats (r = −0.07, *p* = 0.85; Figure 6B). No effect of saline SA was found on [^123^I]IBZM Gamma in RHAs or RLAs.

## 3. Discussion

To our knowledge, this is the first study to concurrently evaluate the effects of a history of cocaine SA on impulsive action, novelty-seeking, and pre- and postsynaptic markers of DA signaling in the striatum. Here, we replicated and extended our previous findings concerning lower D_2/3_R availabilities and a higher AMPH-induced DA release in the striatum in relation to impulsive action and novelty-seeking as an endophenotype predictive of a higher propensity to take cocaine. Furthermore, we showed that a history of cocaine SA did not affect striatal D_2/3_R availabilities or alter impulsivity, but it was associated with the baseline-dependent blunting of stimulated DA release in high-impulsive RHA rats only, as well as a selective baseline-dependent increase in novelty-seeking in low novelty-seeking RLA rats only. Our findings confirm the view that high impulsive action and high novelty-seeking precede and predict a greater rate of cocaine SA, and they indicate that novelty-seeking can also emerge as a result of even moderate drug use in low-novelty seekers. In light of these data, it seems likely that the exaggerated impulsivity [5] and reduced striatal D_2/3_R availability [53] seen in drug abusers predate, and are not consequences of, drug abuse. In contrast, our data indicate that the blunted release of DA observed in drug users (reviews in [53,54]) is likely an adaptive consequence of drug use rather than a pre-existing DAergic abnormality predisposing to drug abuse.

Although there are conflicting studies concerning the predictive value of impulsivity and novelty-seeking on drug abuse, and how these traits are impacted by drug exposure, our findings align with previous studies [17,31,32] which show that both behavioral traits predict higher rates of cocaine intake, even though novelty-seeking can also result from drug use in low novelty-seekers. Unlike novelty-seeking, impulsivity was not affected following cocaine exposure, regardless of the baseline levels of impulsivity. The lack of effect of cocaine exposure on impulsive action is not an isolated finding, and it has been reported using both short- [25] and long-access schedules of cocaine SA [27] in normal impulsive rats. However, it contrasts with previous studies [16,21] that showed a baseline-dependent increase in impulsivity in highly impulsive rats. The reason for this discrepancy is unclear, although it is important to note that these studies did not include a comparative group of operated rats that self-administered the saline-vehicle as a control.

Current research indicates that altered DA transmission in the striatum contributes to impulsivity and novelty-seeking [36], and it may mediate the link with a higher propensity for drug abuse. In accordance with previous findings [16,50,52], we found that impulsivity and novelty-seeking are associated with reduced levels of striatal D_2/3_R, and it confirmed our earlier observation [52] that both behavioral traits are also related to a heightened psychostimulant-induced DA release. This is in line with neuroimaging findings in healthy humans [44,45,46,47], and it supports the notion of DA hyper-responsivity inimpulsivity and novelty-seeking. Furthermore, increasing the activity of midbrain DA neurons via optogenetic stimulation [66], or reducing it via intra-midbrain infusions of a D_2/3_R agonist [67], increases and decreases, respectively, premature responses. Such DA hyper-responsiveness observed in impulsivity may result in the downregulation of the striatal post-synaptic D_2/3_R, which is in line with the observation that baseline striatal D_2/3_R availability negatively correlated with the magnitude of AMPH-induced DA release [52]. The mechanism driving such DA hyper-responsivity may involve the desensitization and/or downregulation of the midbrain D_2/3_R-autoR that mediates inhibitory feedback control over DA neuron excitability and DA release [68], and/or the reduced expression of the DA transporter and subsequent reduced clearance of the released DA in the striatum; as both indexes of DA function have been found to be reduced in impulsive subjects [50,69,70,71,72]. Taken together, these results suggest that hyperdopaminergic activity plays a role in the mechanisms underlying impulsivity and novelty seeking, which may enhance the salience of reward-paired cues, and therefore, it may contribute to the vulnerability for cocaine use.

Following cocaine exposure, we found no alteration in of D_2/3_R availability in the striatum, regardless of baseline levels of impulsive actions. In contrast, there was a blunted striatal DA release in response to AMPH in high-impulsive RHAs, indicating a tolerance to AMPH’s ability to increase striatal DA levels, possibly as a compensatory response to chronic cocaine-induced DA elevations. A tolerance to the DA-elevating effect of cocaine in the nucleus accumbens has previously been observed via microdialysis using both short- [58,59,73] and long-access [74,75] schedules of cocaine SA, and it is viewed as a critical determinant in the development of drug dependence and addiction [76]. Our results extend this effect to the dorsal striatum, where hypodopaminergic functioning has been linked to compulsive drug use [77]. Moreover, we showed that this tolerance was baseline-dependent, and it correlated negatively with baseline levels of AMPH-induced DA overflow, suggesting a floor effect of cocaine exposure in reducing evoked DA release. This finding indicates that cocaine induced a DA hypo-responsivity, specifically in DA hyper-responsive impulsive animals at baseline, which may contribute to their increased propensity to excessive cocaine intake, in order to compensate for the decreased effects of cocaine.

An advantage of using in vivo neuroimaging is its non-invasive and repeatable nature, which allowed us to concurrently study, for the first time, cocaine-induced changes in D_2/3_R availabilities and evoked a DA release in relation to impulsivity and novelty-seeking using a within-subject design. Furthermore, using similar non-invasive neuroimaging methods to measure DA function allowed us to bridge the gap between animal models and clinical research in drug abuse. Indeed, our data indicate that the heightened impulsivity [5], and striatal D_2/3_R deficits [53], consistently reported in drug abusers, are likely risk factors for drug abuse, whereas their blunted DA response to psychostimulants (reviews in [53,54]) likely results from chronic drug use. Moreover, our results may help to shed light on a long-standing question in drug abuse research, which concerns whether drug abuse fundamentally results from too little, or too much, DA signaling. According to one theory, increased susceptibility to drug abuse reflects an innate decreased DA reward system responsiveness that drives individuals to use drugs to overcome this DA deficiency [78]. A contrasting theory is that increased susceptibility to drug abuse results from an increased responsiveness of the DA reward system, which confers an increased motivation to initiate drug use [79], which, in turn, further sensitizes DA responses to drugs, leading to enhanced drug intake via incentive–motivational processes [80,81]. Based on our data, we propose an integrative model that encompasses both processes, and an increased (i.e., sensitization) and a decreased (i.e., tolerance) DA responsiveness, but at different temporal phases of the drug abuse process, as in the opponent–process model [82,83]. Individuals at risk for drug abuse initially exhibit an innate hyper-responsive DA system to reward and reward-associated cues, promoting impulsive actions and novelty-seeking behaviors, and producing stronger initial drug-reinforcing effects that would contribute to the perpetuation of drug use. However, after repeated drug use, tolerance to drug-induced striatal DA elevations would gradually develop, leading to a compensatory increase in drug consumption to overcome the reduced rewarding effects. This alternative model reconciles the two theories into a dynamic framework in which sensitized and tolerant DA responses would occur sequentially, as pre-existing risk factors and consequences of drug abuse, respectively, which ultimately promote compulsive drug use.

Although informative, the short-access schedule of reinforcement used here does not capture the specific hallmarks of the addiction process, such as persistent drug use despite negative consequences, loss of control of drug consumption, and escalation of drug use [84]. However, short-access to cocaine SA proved sufficient to recapitulate the predictive value of impulsivity and D_2/3_R deficits for cocaine consumption [16] and the tolerance-related blunting of DA responses [85] previously evidenced using an extended-access procedure to cocaine SA. Future studies are needed to investigate whether RHA rats are more susceptible to developing DSM-like features that are relevant to addiction in rats [84] using SA paradigms that better model human patterns of cocaine intake, and that are more effective than the short-access model used here at producing addiction-like behaviors in rats, such as the extended- [86] or intermittent-access models [87].

As a further limitation, our study included male rats only, thus potentially limiting the generalizability of the results to females. Only a handful of studies have directly compared males and females concerning impulsive action and susceptibility to drug abuse. There is some evidence suggesting a sex-dependent hormonal influence on impulsive action, and that its association with drug abuse might be stronger in females than in males [88]. Future studies should therefore evaluate sex differences in impulsivity as predictors of drug abuse.

In addition to impulsivity and novelty-seeking, the trait of anxiety has also been associated with drug abuse [89]. In this regard, RHA rats have been shown to display lower levels of anxiety and emotionality, as well as a proactive coping style and a lower response in the hypothalamo-pituitary-adrenal axis to stress when compared with RLAs [90]; however, they are still more vulnerable to cocaine intake than RLAs. Interestingly, and in parallel with observations made in Roman rats, rats classified as high sensation seekers, based on their locomotor reactivity in a novel inescapable environment, also display reduced anxiety compared with low sensation seekers [91]; but still, they are also more vulnerable to cocaine intake [20]. Altogether, these studies suggest that impulsivity and/or novelty/sensation seeking might prevail over anxiety-related behaviors to predict a higher vulnerability to drug abuse.

In summary, we showed that highly impulsive rats exhibited low availabilities of D_2/3_R, and high AMPH-induced DA release in the striatum, which predisposes them to high cocaine intake. Critically, although impulsivity and D_2/3_R availabilities failed to vary robustly following cocaine intake, irrespective of their baseline levels, a history of cocaine attenuated AMPH-induced DA release in a baseline-dependent manner in highly impulsive rats only, which may reveal a potential mechanism underlying the development of compulsive drug taking in vulnerable individuals. These findings augment a growing literature demonstrating the impact of individual differences, in impulsive action and novelty-seeking, on DA function, susceptibility to drug use, and adaptive consequences potentially involved in the transition from controlled consumption to compulsive drug use [16,29,92].

## 4. Materials and Methods

### 4.1. Animals

Male RHA and RLA rats, at three months old, and weighing 275–300 g, were used from our breeding colony of outbred lines of Roman rats at the University of Geneva. Rats were pair-housed under temperature-controlled conditions, and they were maintained under a reversed 12 h light–dark cycle, with lights turning off at 7:00 a.m. Animals were food-restricted to 85–90% of their free-feeding weight throughout the experiment, but water was provided ad libitum. Experiments were conducted in accordance with Swiss Federal Law on animal care, and they were approved by the Animal Ethics Committee of the Geneva Canton.

### 4.2. General Procedure Animals

**Figure 7:**
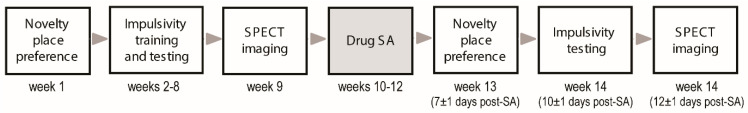
Timeline of the experiment.

As shown in Figure 7, rats (n = 27/line) were first tested using the novelty-induced place preference test. Rats were then trained and tested for impulsivity in the five-choice serial time task. Part of the cohort (n = 18 rats/line) was then scanned using single photon emission computed tomography (SPECT) and the D_2/3_R antagonist radiotracer [^123^I]IBZM, to index striatal D_2/3_R availability and amphetamine- (AMPH-) induced DA release at baseline. Subsequently, rats from the two lines were randomly subdivided into two groups and trained to self-administer either cocaine (n = 15 rats/line) or saline (n = 12 rats/line) for 14 days. All rats were then re-tested for novelty-seeking and impulsivity, and part of the cohort underwent a second [^123^I]IBZM SPECT scan.

### 4.3. Novelty-Induced Place Preference Test (NIPP)

The apparatus consisted of open fields (ActiMot, TSE Systems, Bad Homburg, Germany) which were divided into two equally sized compartments (23 × 46 × 40 cm) using a sliding door. These compartments differed in color, patterned walls, and floor texture. During the first 4 days, rats were placed into one compartment (i.e., a familiar compartment) for 15 min/day, with the door between compartments closed. On the fifth day, rats were placed into the familiar compartment but with the door opened, allowing rats to move freely between compartments for 10 min. Novelty-seeking was assessed using the percentage of total time spent in the novel compartment vs. the familiar compartment.

### 4.4. Five-Choice Serial Reaction Time Task (5-CSRTT)

Rats were trained with the 5-CSRTT in 11 modular operating chambers (Med Associates Inc., St. Albans, VT, USA). Each chamber contained a house-light and was equipped with a curved wall, including five equally spaced response holes that were raised 2.5 cm from the floor. Each of the response holes was equipped with a yellow cue light and head-entry detectors. The opposite wall was equipped with a food receptacle containing a light, and it was connected to an external dispenser. At the beginning of each session, the house light was illuminated and a food pellet (45 mg purified rodent tablets, TestDiet, Sandown Scientific, Sawbridgeworth, UK) was delivered. By collecting the pellet, the rat initiated the first trial of the session. After an inter-trial interval (ITI) of 2 s, a light stimulus appeared pseudo-randomly in one of the 5 response holes for a period of 30 s. A nose-poke into the correct location of the visual stimulus was rewarded with a pellet. A nose-poke into any other response hole counted as incorrect and was punished with a time out (TO), period of 5 s, during which, the house light was extinguished, and no food was delivered. Missed trials (i.e., omissions) were also recorded, and they resulted in a TO period. Responses made before the onset of the visual stimulus during the ITI were considered premature, they resulted in a TO period, and subsequent resetting of the trial. Throughout the training period, and with improvement of the animal performances, the level of difficulty progressively increased by shortening the light stimulus duration from 30 s to 1.5 s, shortening the time available for the rat to respond (limited hold), from 30 s to 5 s, and increasing the duration of the ITI, from 2 to 7 s, over 8 training phases. The criteria for level change (i.e., more than 80% accuracy and less than 30% omissions), were to be maintained over 2 consecutive sessions. Rats were trained until reaching the final set of task parameters (stimulus duration, 1.5 s; limited hold, 5 s; ITI, 7 s) fulfilling the criterion performance (>80% accuracy and <30% omissions). Once rats achieved stable performances across 3 consecutive days (<10% variation in accuracy), rats were challenged with a 100 trial session, in which the ITI was increased to 9 s to increase the number of premature responses. Behavioral performance was assessed, as follows [93]: premature responses, [#premature/(#correct + #incorrect + #omission)] × 100, to measure impulsive action; choice accuracy, [#correct/(#correct + #incorrect)] × 100, to measure attentional function; omissions[#omissions/#trials] × 100, to measure attention, motivation, and motor ability; the latency to collect food rewards as a measure of motivation; and the latency for a correct response as a measure of processing speed and motor ability.

### 4.5. SPECT Imaging

Rats (n = 18 rats/line) were scanned using the U-SPECT-II imaging system (MiLabs, Utrecht, The Netherlands) and [^123^I]IBZM, as previously described [94]. Briefly, rats were anaesthetized with 2.5% Isoflurane, and positioned into a custom built head-holder (MiLabs, Utrecht, The Netherlands) to ensure the reproducible positioning of the animal’s head within the field of view of the SPECT system. Rats were injected i.v. with [^123^I]IBZM, and they were scanned for 134 min. There was no significant between-line difference in the doses of [^123^I]IBZM injected in Pre-SA and Post-SA conditions (RHA: pre-SA = 33.8 ± 5.1 MBq and post-SA = 34.5 ± 6.7 MBq; RLA: pre-SA = 36.1 ± 4.7 MBq and post-SA: 35.8 ± 4.7 MBq; Line effect: F_1,34_ = 1.49, *p* = 0.231; Time effect: F_1,34_ = 0.04, *p* = 0.851; Line × Time: F_1,34_ = 0.30, *p* = 0.589). AMPH (1.5 mg/kg; i.v) was injected via a 72 min post-radiotracer injection. The first (0–72 min) and second (73–134 min) portion of SPECT acquisition measured [^123^I]IBZM kinetics at baseline, and in response to AMPH, respectively. Body temperature was monitored during the scan, and it was maintained at 37 ± 1 °C using a thermostatically controlled heating blanket.

Reconstructed [^123^I]IBZM images were analyzed, as previously described [94], using PMOD V3.9 software (PMOD Technologies, Zurich, Switzerland). Briefly, SPECT images were co-registered with a magnetic resonance imaging (MRI) rat brain template [95]. A region of interest (ROI) template was created that consisted of fixed-size circles (2 mm diameter) placed bilaterally on the DST (herein the caudate-putamen is located), VST (herein the nucleus accumbens is located), and a single ellipse on the cerebellar cortex. To minimize partial volume effects, ROIs were placed on the central planes where the structures appeared. The ROI template was defined using the MRI template, and it was applied to the co-registered SPECT images to produce time–activity curves (TACs) for the target-rich (DST and VST) and reference (cerebellum) regions. Nonlinear least squares fitting (NLSF) analyses, based on the linear extension of the simplified reference region model LSRRM [96] were applied to the TACs to estimate the non-displaceable binding potential (BP_ND_) as an index of D_2/3_R availability, and Gamma as an index of AMPH-induced DA release in DST and VST. Briefly, the LSSRM considers temporal disturbances in radioligand specific binding caused by drug-induced changes in the levels of endogenous DA during a single-scan session [96]. The LSSRM assumes that a stable physiological state is disrupted at some point in the experiment, and it allows the rate of the radioligand, from the receptor, k2a, to change over time in response to a local variation in DA levels (k_2a_ = k_2_/[1 + BP_ND_]), where k_2_ is the tissue-to-plasma efflux constant in the target region. Changes in BP_ND_ in competition studies are assumed to reflect inverse variations in the concentration of extracellular DA [97]. Increased competition between DA and the radioligand for receptor binding is reflected by a temporal change in k_2a_, which is accounted for by a time-dependent parameter, k_2a_ + Gamma × h(t), where Gamma represents the magnitude of the radioligand displacement, and the function h(t) describes a rapid change in radioligand binding after the onset of competition and its dissipation over time. The decay function h(t) = exp[−τ(t − T)] describes temporal fluctuations in the model parameters, where τ controls the rate at which the competition effects die away and T represents the time of competition onset. Therefore, an increase in k_2a_, reflected by a reduction in BP_ND_, and caused by an increase in AMPH-induced DA release, results in a positive value of Gamma. Here, T was set to the time of an AMPH injection, and τ was set to 0 as the reduction in BP_ND_, caused by AMPH administration, is long lasting and is unlikely to recover during the 60 min when post-AMPH SPECT measurements were taken in our study. Indeed, previous studies showed that the decrease in BP_ND_ of D_2/3_R radioligands, such as [^11^C]raclopride [98], [^123^I]IBZM [99] and [^11^C]-(+)-PHNO [100], is sustained for at least 3 h following AMPH administration.

Standard errors for BP_ND_ and Gamma, as estimated during the NLSF procedure, was given by the diagonal of the measurement error covariance matrix, expressed in a percentage of the parameter value (percent standard error, %SE), and it was used to assess the parameter identifiability [101]. A smaller %SE indicates better identifiability.

### 4.6. Intravenous Self-Administration

Rats were implanted with a jugular catheter, with the distal end connected to a mid-scapular vascular access button (Instech Laboratories, Markkleeberg, Germany). After recovery, rats were placed in operant chambers (Med Associates Inc., St. Albans, VT, USA) and they were allowed to self-administer either cocaine (0.4 mg/kg/infusion; University Hospital of Geneva Pharmacy) or a saline vehicle, under a fixed-ratio-1 schedule of reinforcement during 2 h daily sessions for 14 days, as previously described [62]. Each chamber contained a house light and two nose-poke ports, each equipped with a cue light, and whose position was counterbalanced across rats. During the SA session, nose-poking into the active port resulted in an infusion of saline or cocaine, which was followed by a 20 s TO period. Responses into the inactive port were recorded, but no consequences followed. For ethical reasons, the total drug intake was limited to 60 infusions/session.

### 4.7. Statistical Analysis

Homogeneity of variances and normal data distributions were verified using Levene’s and Shapiro–Wilk’s tests, respectively. Between-line comparisons at baseline were performed using two-tailed independent Student’s *t*-tests for normal data, and Mann–Whitney U-tests for non-normal data. Drug infusions earned throughout the 14 sessions of SA were subjected to Kruskal–Wallis ANOVA, followed by Mann–Whitney U-tests. The mean number of drug infusions self-administered during the last days of SA were compared using two-way ANOVA with treatment (saline vs. cocaine), and lines (RHA vs. RLA) as between-subject factors.

To reveal the impact of cocaine SA on behavioral performances and SPECT imaging data, a repeated measures general linear model (RM-GLM) ANOVA was performed with time (pre-SA vs. post-SA) as within-subject factor, and treatment (saline vs. cocaine) and line (RHA vs. RLA) as between-subject factors. Significant effects were analyzed with paired or unpaired post hoc Student’s *t*-tests, where appropriate. Spearman’s rank correlations were used to assess the associations between cocaine-induced changes in novelty-seeking ((post-cocaine − baseline)/(baseline) × 100), baseline novelty-seeking, and cocaine-induced changes in [^123^I]IBZM Gammas ((post-cocaine − baseline)/(baseline) × 100) and baseline [^123^I]IBZM Gammas. Statistical significance was set at *p* < 0.05. Data were analyzed using SPSS statistics (IBM, version25.0).

## Figures and Tables

**Table 1:** Behavioral performances of RHA and RLA rats on the NIPP and 5-CSRTT before (Pre-SA) and following (Post-SA) cocaine or saline SA.

	Cocaine				Saline			
	Pre-SA		Post-SA		Pre-SA		Post-SA	
	RHA (n = 15)	RLA (n = 15)	RHA (n = 15)	RLA (n = 15)	RHA (n = 12)	RLA (n = 12)	RHA (n = 12)	RLA (n = 12)
**NIPP**								
%time in novel compartment	63.5 ± 2.5	32.2 ± 3.4 **	56.5 ± 3.9	50.6 ± 3.2 ^††^	60.0 ± 3.0	39.9 ± 3.5 **	58.9 ± 4.3	41.7 ± 3.0 **
**5-CSRTT**								
%Premature	53.4 ± 5.0	21.5 ± 3.5 **	30.5 ± 3.3	15.3 ± 2.7 **	47.7 ± 3.6	17.8 ± 2.5 **	34.7 ± 3.5	12.9 ± 2.3 **
%Accuracy	83.8 ± 1.3	94.2 ± 1.0 **	88.0 ± 1.4	94.7 ± 1.1 **	82.7 ± 1.1	94.5 ± 1.2 **	83.4 ± 1.5	91.9 ± 1.6 **
%Omission	16.1 ± 1.7	24.9 ± 1.8 **	15.0 ± 1.5	28.7 ± 2.4 **	17.8 ± 2.4	25.0 ± 2.6 *	11.7 ± 1.7	26.1 ± 2.3 **
Tendency to correct (s)	0.87 ± 0.02	0.89 ± 0.03	0.85 ± 0.02	0.91 ± 0.03	0.87 ± 0.04	0.93 ± 0.05	0.81 ± 0.02	0.95 ± 0.06
Tendency to seek reward (s)	1.36 ± 0.05	2.40 ± 0.14 **	1.29 ± 0.04	2.24 ± 0.09 **	1.41 ± 0.05	2.06 ± 0.1 **	1.44 ± 0.06	2.33 ± 0.1 **

Data are mean ± SEM. Significantly different from RHA rats at * *p* < 0.05 and ** *p* < 0.001. Significantly different from the respective pre-SA condition at ^††^ *p* < 0.001.

## Data Availability

The data presented in this study are available on request from the corresponding authors.

## Supplementary Materials

The following supporting information can be downloaded at: https://www.mdpi.com/article/10.3390/ijms241713238/s1.
