# Supplementary material for: Repeated Cocaine Intake Differentially Impacts Striatal D2/3 Receptor Availability, Psychostimulant-Induced Dopamine Release, and Trait Behavioral Markers of Drug Abuse
Source: Int J Mol Sci. 2023 Aug 26;24(17):13238. doi: 10.3390/ijms241713238 (PMC10487888; doi:10.3390/ijms241713238)
Supplement: Supplementary file 1 [file ijms-24-13238-s001.zip › ijms-2579516-supplementary.pdf]

Table S1: In Vivo Binding of [^{123}I]IBZM in the dorsal (DST) and ventral (VST) striatum of RHA and RLA rats at baseline.

	RHA (n=18)	RLA (n=18)
**DST**		
BP_{ND}	1.84 ± 0.41*** (%SE = 2.23 ± 0.67)	2.86 ± 0.38 (%SE = 3.14 ± 1.34)
Gamma	0.036 ±0.008*** (%SE = 22.0 ± 5.8)	0.016 ±0.004 (%SE = 25.1 ± 4.0)
**VST**		
BP_{ND}	1.16 ± 0.29*** (%SE = 2.52 ± 0.74)	1.706 ± 0.25 (%SE = 3.51 ± 1.52)
Gamma	0.013 ±0.011 (%SE > 100 ± 100)	0.008 ±0.008 (%SE > 100 ± 100)

*%SE represents the mean percent standard error of BP_{ND} and Gamma values, as given by nonlinear least squares fittings of [^{123}I]IBZM kinetics using the linear extension of the simplified reference region model, and it was used to assess parameter identifiability. Data are mean ± SD. Significantly different from RLA rats at ***p < 0.001.*
